# The relationship between alexithymia, sensory phenotype and neurophysiological parameters in patients with chronic upper limb neuropathy

**DOI:** 10.1007/s00702-020-02282-z

**Published:** 2020-12-14

**Authors:** Gianluca Isoardo, Stefano Ciullo, Paolo Titolo, Elena Fontana, Bruno Battiston, Maurizio Stella, Nicola Luxardo, Federica Laino, Giuseppe Migliaretti, Ilaria Stura, Rita B. Ardito, Mauro Adenzato

**Affiliations:** 1 Department of Neurosciences and Mental Health, Hospital “Città della Salute e della Scienza di Torino”, Turin, Italy; 2 Department of Psychology, University of Turin, Turin, Italy; 3 Department of Orthopedics and Traumatology, UOD Reconstructive Microsurgery, Hospital “Città della Salute e della Scienza di Torino”, Turin, Italy; 4 Department of Plastic Surgery Burn Center, Hospital “Città della Salute e della Scienza di Torino”, Turin, Italy; 5 Department of Anesthesia, Intensive Care and Emergency, Unit of Pain Management and Palliative Care, Hospital “Città della Salute e della Scienza di Torino”, Turin, Italy; 6 Department of Public Health and Paediatric Sciences, University of Turin, Turin, Italy; 7 Department of Neuroscience “Rita Levi Montalcini”, University of Turin, Via Cherasco 15, 10126 Turin, Italy

**Keywords:** Alexithymia, Quantitative sensory testing, Neuropathic pain, Carpal tunnel syndrome

## Abstract

In this study, we investigated the relationship between sensory abnormalities evaluated by quantitative sensory testing (QST) and alexithymia, depression and anxiety in patients with neuropathic pain involving the upper limbs. We enrolled 62 patients (34 with carpal tunnel syndrome, 7 with brachial plexopathy, 3 with cervical painful radiculopathy, 5 with ulnar entrapment neuropathy at elbow and 13 with post-burn hypertrophic scars) and 48 healthy controls. All underwent nerve conduction studies (NCS), evaluation of cold, heat pain and vibration detection threshold (VDT) by QST and evaluation of alexithymia by Toronto Alexithymia Scale (TAS-20), depression by Beck Depression Inventory II (BDI-II), anxiety by State-Trait Anxiety Inventory (STAI-Y), level of psychological distress by 12-item General Health Questionnaire (GHQ-12) and perceived social support by the Multidimensional Scale of Perceived Social Support (MSPSS). The general linear model analysis revealed a significant relationship between TAS-20 overall and TAS-20 sub-score for difficulty identifying feelings and VDT z-scores in the left index with no interaction by year of education and sensory NCS results. Our results demonstrated the association between impairment of vibratory sensation of the left hand, reflecting cutaneous mechanoceptor dysfunction, and alexithymia, particularly the difficulty to identify feelings. The importance of delivering to patients with neuropathic pain personalized care that takes into account not only the neurophysiological aspects but also the aspects of mental functioning is discussed.

## Introduction

The International Association for the Study of Pain (IASP) defined pain as “an unpleasant sensory and emotional experience associated with actual or potential tissue damage, or described in terms of such damage” (IASP 2020). Mood and behavioral changes have been described in patients with chronic pain, and modulation of neural circuits involved in motivation regulation is critical for translation between acute/subacute and chronic pain (Baliki and Apkarian 2015; Vachon-Presseau 2016). A biopsychosocial approach to the study and management of pain involves the identification of the inter-relationships between neurobiological mechanisms, concurrent behavioral and psychological manifestations, and environmental factors (Meints and Edwards 2018). Depression, anxiety and emotional distress are frequent in patients with chronic pain and are critical to long-term outcomes (Turk et al. 2010; Meints and Edwards 2018).

Alexithymia (from Greek *a*: loss, *lexis*: word, *thymia*: mood or emotion) is a personality construct characterized at an intrapersonal level by an impairment in identifying feelings, inability to find appropriate words to describe them, restricted imagination with a lack of fantasy and heightened preoccupation with the external details of events (Sifneos 1973; Lumley et al. 2007; Lane et al. 2015; Di Tella and Castelli 2016; Keefer et al. 2019; Di Tella et al. 2020). Described initially in patients with classic psychosomatic diseases (Sifneos 1973), substance abuse and posttraumatic stress (Lumley et al. 2007; Keefer et al. 2019), alexithymia has subsequently been characterized as a major risk factor for depression and anxiety (Lumley et al. 2007).

The possible role of alexithymia in chronic pain has been addressed in previous studies, demonstrating a variable association with pain intensity (Hosoi et al. 2010; Makino et al. 2013), but a strong association with depression, anxiety, maladaptive early schemas (Saariaho et al. 2015; Di Tella and Castelli 2016) and catastrophising (Makino et al. 2013). The majority of studies evaluated the presence of alexithymia in patients with chronic primary pain, such as fibromyalgia (Di Tella et al. 2018), complex regional pain syndrome (Margalit et al. 2014) and temporomandibular joint disorder (Nicholas et al. 2019), as well as in patients with chronic secondary musculoskeletal pain (Perrot et al. 2019).

Chronic neuropathic pain is defined as being caused by a lesion or disease of the somatosensory system (Finnerup et al. 2016; Scholz et al. 2019). Identification of sensory signs, both positive (allodynia) and negative (sensory loss), in a neuroanatomically plausible distribution is a critical step in grading the certainty of a diagnosis of neuropathic pain (Finnerup et al. 2016). Quantitative sensory testing (QST) has been recognized as a reliable and reproducible tool for the assessment of somatosensory abnormalities in patients with neuropathic pain (Geber et al. 2011; Backonja et al. 2013). QST has been extensively investigated as a potential biomarker for the diagnosis of neuropathic pain, subgrouping of patients in clinical trials, and outcome predictions following treatment (Smith et al. 2017; Treede 2019; Rosenberger et al. 2020). Furthermore, the definition of the association between abnormalities of different sensory modalities, i.e. the sensory phenotype, may point to a possible underlying pathophysiology of pain (Treede 2019).

Analyses of the correlation between sensory impairment and psychological abnormalities have rarely been carried out in patient with neuropathic pain. Baron et al. (2017) for instance found depressive symptoms more frequently associated with a sensory phenotype defined by prominent sensory loss. However, to our knowledge, no previous studies investigated the relationship between neuropathic pain, alexithymia and sensory phenotype. To this end, in this study, we evaluated the relationship between sensory phenotype and alexithymia in patients with neuropathic pain in the upper limbs.

## Materials and methods

### Participants

The study was approved by the ethic committee of the hospital Citta’ della Salute e della Scienza di Torino. Patients and controls gave their informed consent to participate the study. Sixty-two patients with neuropathic pain involving at least one hand and 48 healthy controls were enrolled. We included patients with the following diseases known to cause chronic (lasting more than 3 months) peripheral neuropathic pain (Finnerup et al. 2016; Scholz et al. 2019; Schug et al 2019): carpal tunnel syndrome (CTS), brachial plexopathy (BP), painful cervical radiculopathy (PR), ulnar neuropathy at elbow (UNE), post-burn hypertrophic scars (PBHS). The diagnosis of CTS was based on the clinical and neurophysiological criteria set forth by the American Academy of Neurology (1993) and the American Association of Electrodiagnostic Medicine (Jablecki et al. 2002). BP was diagnosed on the basis of electrodiagnostic (Ferrante 2004) and/or MRI findings of brachial plexus disease. Root avulsion (RA) was diagnosed by MRI evaluation or direct surgical exploration (Wade et al. 2018). PR was diagnosed according to Scholz et al. (2019), UNE was diagnosed according to Mondelli et al. (2005) and PBHS were defined as previously described (Isoardo et al. 2012). Exclusion criteria were: age lower than 14 years and higher than 80 years; inability to complete the QST examination with sufficient accuracy; history of alcohol or drug abuse; family or past history, clinical or laboratory evidence of cervical central nervous system diseases or polyneuropathy involving lower limbs.

All patients (34 with CTS, 7 with BP, 3 with cervical PR, 5 with UNE and 13 with PBHS) underwent a full clinical evaluation, including a Medical Research Council (MRC) scale score (Kendall et al., 2005) of their abductor pollicis brevis (APB) and abductor digiti minimi (ADM) and pinprick, touch and position sense assessments of both upper limbs. In addition to evaluating pinprick and touch sense, the pain sites were also assessed for signs of allodynia in response to brushing, as part of the DN4 questionnaire (Bouhassira et al. 2005). Self-reported mean pain intensity in the week before examination was graded on an 11-point numerical rating scale (NRS), with scores ranging from 0 (no pain) to 10 (worst possible pain) (Jensen and McFarland 1993).

### Nerve conduction studies (NCS)

Participants underwent bilateral motor NCS of the median and ulnar nerves and antidromic sensory NCS of the median, radial and ulnar nerves, according to standard techniques (Isoardo et al. 2012). Sensory NCS of median nerve were recorded from the index finger and of the ulnar from the little finger. In patients with BP/RA, PR and UNE, sensory NCS of the antebrachial lateral (Goslin and Krivikas 1999) and medial cutaneous nerves (Seror 2002) were also performed. Comparison of both the antidromic median and ulnar sensory latency to the fourth digit was performed bilaterally in patients with PBHS and in participants with clinical suspicion of CTS who had normal motor and sensory conduction in the median nerve (Preston 1999; Jablecki et al. 2002). A median sensory latency at least 0.5 ms higher than the ulnar latency at the fourth digit was considered suggestive of CTS (Preston 1999). A needle examination was performed in all patients with BP/RA and CTS. NCS and needle examinations were performed with commercially available electrodiagnostic equipment (Viking Quest, Carefusion, Wisconsin).

### Quantitative sensory testing (QST)

QST was performed to evaluate the thresholds for perception of cold (CDT), heat-induced pain (HPT), and vibration (VDT). CDT, HPT and VDT were evaluated at the palmar surface of the index and little finger; CDT and HPT were also evaluated at the dorsum of the hand. VDT, CDT and HPT were evaluated with a commercially available thermal and vibratory stimulation device (Medoc TSA II, Durham, North Carolina). HPT was evaluated by the method of limits (Isoardo et al. 2012). Stimulation began at 32 °C and increased by a rate of 1 °C per second, until the participant perceived a change from a heat sensation to pain, or until the temperature of the probe reached 50 °C. Five trials at each site were averaged to evaluate the HPT. CDT was evaluated using a staircase method with null stimulations (Isoardo et al. 2012). Briefly, three ranges of cooling steps are presented, beginning with a gross 3 °C decrease in temperature. Stimulation began at 32 °C. In this reaction-time-independent evaluation, the participant was asked to define whether or not if s/he had perceived the cooling step. VDT was evaluated by the method of levels with null stimulations. Stimulation began at 0 µm. The participant was asked whether or not if s/he had perceived the vibration step. Thresholds for CDT and VDT were assessed using a computerized algorithm. QST was considered insufficiently accurate if participants failed to identify at least two of five null stimuli during CDT and/or VDT evaluations.

Hypoesthesia for cold and vibration was defined if z-scores for CDT were lower than -2.58, for VDT higher than 2.58. For Heat pain hypoesthesia was defined if no pain was perceived at 50 °C in all trials for each site. Allodynia for heat pain was defined if z-scores were lower than -1.64. Sensory profile was defined pooling data from QST and clinical evaluation of DN4 questionnaire at painful sites. According to Maier et al (2010), loss of thermal or pain (either heat or pinprick) sense was labeled as L1, loss of vibration or touch sense as L2 and combined loss of thermal/pain and vibration/touch as L3, and no loss of thermal/pain and vibration/touch as L0. Allodynia to heat was labeled as G1 and allodynia for mechanical stimulation as G2.

### Psychological, health quality and social support evaluation

Alexithymia was assessed by means of the validated self-report questionnaire 20-item Toronto Alexithymia Scale (TAS-20) (Bagby et al. 1994a, b). The TAS-20 consists of three subscales that investigate the following factors: (F1) difficulty in identifying feelings; (F2) difficulty in describing and communicating feelings; (F3) externally oriented thinking. The total score on the questionnaire allows for the categorization of subjects according to their alexithymic dimension: non-alexithymic (score = 20–51), borderline alexithymic (score = 52–60), or alexithymic (score ≥ 61).

The presence of depressive symptoms was assessed using the Beck Depression Inventory-II (BDI-II). The BDI-II is a 21-item validated self-report instrument assessing depressive symptoms over the previous 2 weeks (Beck et al. 1996). The total score was obtained considering all items, and rated from 0 to 3. A total score of 0–13 indicates minimal depression, 14–19 mild depression, 20–28 moderate depression, and, finally, scores over 29 are indicative of severe depression.

The Y form of the State-Trait Anxiety Inventory (STAI-Y) was used to assess the presence of anxiety symptoms (Spielberger et al. 1983). This self-report questionnaire is divided into two sections, each consisting of 20 items that are scored using a 4-point Likert-type scale: the STAI-Y1 assesses current feelings of apprehension and tension (state anxiety), while the STAI-Y2 evaluates persistent anxiety traits (trait anxiety). Each section has a total score ranging from 20 to 80, with higher scores indicating greater anxiety.

The 12-item General Health Questionnaire (GHQ-12) is a self-report questionnaire assessing levels of psychological distress (Goldberg and Williams 1988). Items are scored on a 1–4 Likert scale, which are subsequently converted into binary scores (i.e., scores 0 or 1 = 0, and scores 2 or 3 = 1, giving a maximum score of 12). Higher scores indicate greater levels of psychological distress with a cut-off set to ≥ 4.

The Multidimensional Scale of Perceived Social Support (MSPSS) is a validated self-report measure of subjectively assessed social support (Zimet et al. 1988). It provides assessment of three sources of social support: family, friends, and significant other. The MSPSS consists of 12 items, each scored on a 7-point Likert scale. The maximum score is 84; higher scores indicate greater perceived support.

### Statistical analysis

Results are expressed as mean ± standard deviation (SD). The normality of the quantitative parameters distribution was analyzed using the Kolmogorov–Smirnov test, and the QST parameters that were non-normally distributed were log-transformed to be analyzed using parametrical methods of inferential analysis (Rolke et al. 2006; Isoardo et al. 2012). Patients’ CDT, HPT and VDT z-scores at each site were calculated as follows:

$$(\log /\ln {\text{ patient value}} - {\text{mean}}\;\log /{\text{ln}}\;{\text{healthy}}\;{\text{control}}\;{\text{values}})/{\text{SD}}\;\log /{\text{ln}}\;{\text{healthy}}\;{\text{control}}\;{\text{values}}$$

Analyzing data from patients with bilateral pain may overstate statistical significance if the comparison is only made for hands (Padua et al. 2005). Since pain frequently affects both hands in our series, to avoid this bias, we decided to perform the statistical analysis both for the hand and for the patients (Padua et al. 2005). The differences among the groups of hands/patients (with painful hands, with non-painful hands and healthy) were analyzed using a Mann–Whitney or Wilcoxon test. Correlations were analyzed by estimating the parametric r-Pearson correlation coefficient. General linear models (GLM) were used to test correlations both dividing for group or for class. Categorical data were compared using a Chi-square test or Fisher’s exact test when appropriate. Statistical analysis was carried out using Statistical Package for the Social Sciences software version 9.0 (SPSS Inc., Chicago, IL, USA). In all analyses, *p*-values < 0.05 were considered as statistically significant. The available sample size of 62 patients allows the study to highlight a difference of at least 10% between pathological compared to normal of TAS-20 score, reaching a Power of 80% with a two-tailed alfa error of 0.05. The hypothesized 10% difference between patients with pain and healthy controls was based upon the results reported by Makino et al. (2013).

## Results

### Demographic and clinical features

Thirty-four patients had CTS (21 bilateral, 7 right, 6 left), 7 BP (3 with concurrent RA; 2 right, 5 left), 3 PR C5-C6 (1 bilateral, 1 right, 1 left), 5 UNE (2 right, 3 left) and 13 PBHS. No difference between patients and healthy controls was noted either for age (52.7 ± 13.6 vs 48 ± 15.5 years) or sex (38 women, 24 men vs 30 women, 18 men). The data of patients with BP, PR and UNE were pooled and considered as other neuropathic pain of the upper limb (ONP). Years of education were significantly lower in patients than healthy controls (11.3 ± 3.5 vs 14.3 ± 4.7 years, *p* = 0.01), due to a significantly higher number of patients with primary (5 vs 1 healthy control) and secondary school licenses (18 vs 7 healthy controls) and a lower number of graduates (9 vs 19 healthy controls). In patients,  APB and ADM MRC scores were lower than in healthy controls both on the right and left side (respectively,  APB right = 4.2 ± 1.3, left = 3.5 ± 1.9; ADM right = 4.3 ± 1.2 and left = 4.1 ± 1.6 vs 5 ± 0 in APB and ADM, p < 0.0001). Neuropathic pain involved hands bilaterally in 30 patients, the right hand in 16 and the left hand in 16. All patients and controls were right handed. The mean NRS was 6.2 ± 2.4 and DN4 was 5.4 ± 2.2, and did not differ among CTS, PBHS and ONP (CTS NRS = 5.7 ± 2.7, DN4 = 5.1 ± 2.4; PBHS NRS = 6.3 ± 1.4, DN4 = 6 ± 2.8; ONP NRS = 7.5 ± 1.7, DN4 = 6.1 ± 1.7; Kruskal–Wallis NRS *p*: 0.07, DN4, *p*: 0.244). Itching, burning pain and mechanical allodynia were significantly more frequent in PBHS (respectively, in 11, 10 and 8 of 13 patients, respectively, *p*: 0.002, *p*: 0.01, *p*: 0.007), tactile and pinprick hypoestesia were significantly more frequent in ONP (both in 12 of 15 patients, respectively, *p*: 0.003 and p: 0.002) and tingling was significantly more frequent in CTS (in 30 of 34 patients, *p*: 0.03).

### Nerve conduction studies

Results of sensory NCS are summarized in the Table 1. The amplitude of bilateral median and ulnar sensory action potentials (SAPs) and median sensory conduction velocity (SCV) were significantly lower in patients than healthy controls. Painful and non-painful hands had lower median SCVs and median and ulnar SAP amplitudes than healthy controls’ hands (median SCV painful hands = 47.5 ± 9.7 m/s, non-painful hands = 48.4 ± 6.7 m/s, healthy controls’ hands = 60.7 ± 7.7 m/s; median SAP amplitude painful hands = 25.2 ± 17, non-painful hands = 30 ± 25.3, healthy controls hands = 53.1 ± 21.5 µV; ulnar SAP amplitude painful hands = 30.6 ± 17.2, non-painful hands = 28.4 ± 20.2, healthy controls’ hands = 51.1 ± 22.1 µV, all comparison *p* < 0.001). No difference was noted between painful and non-painful hand for median (*p* = 0.63) and ulnar SCV (*p* = 0.91), median (*p* = 0.62) and ulnar SAP amplitude (*p* = 0.24). The median motor conduction velocity was significantly lower in patients than in healthy controls (respectively, right = 52.7 ± 4.7 vs 57.9 ± 6.3; left = 52.9 ± 5.2 vs 59.6 ± 5 m/s *p* < 0.01) and median motor latency was higher in patients than in healthy controls (respectively, right = 4.2 ± 1.1 vs 3.2 ± 0.3; left = 4 ± 1 vs 3.1 ± 0.4 ms *p* < 0.0001) (see Table 2).

**Table 1:** Summary of sensory nerve conduction studies results

	Median nerve				Ulnar nerve			
	SCV (m/s)		SAP amplitude (µV)		SCV (m/s)		SAP amplitude (µV)	
	Right	Left	Right	Left	Right	Left	Right	Left
All patients	47.8 ± 8.8^a^	47.8 ± 9.3^a^	24.7 ± 16.6^a^	28.6 ± 20.4^a^	56.7 ± 7.2	56.9 ± 7.7	28.9 ± 16.8^a^	32.1 ± 18.6^b^
CTS	44 ± 7.2^a^	44.9 ± 8.9 ^a^	21.6 ± 13.9^a^	25.5 ± 16.2^a^	59.4 ± 6.3	59.4 ± 8.1	32.4 ± 16.9^b^	35.8 ± 16.7^c^
PBHS	52.4 ± 6.4^b^	49.3 ± 6.5^d^	23.1 ± 13.8^b^	25.4 ± 11.4^b^	49.6 ± 5.9^b^	50.9 ± 2.1^b^	17.2 ± 11.1^a^	21.9 ± 13.7^b^
Other neuropathic pain	56.9 ± 8.1	57.1 ± 7.6	39.6 ± 23.2	39.7 ± 34.6	53.3 ± 5.2^d^	53.2 ± 3.9	28.7 ± 16.9^d^	28.9 ± 24.3^d^
Healthy controls	59.7 ± 7.8	62.2 ± 7.6	49.7 ± 21.2	57.6 ± 21.9	58.5 ± 6.8	58.3 ± 6.8	52.4 ± 22.3	52 ± 22.8

*CTS*  carpal tunnel syndrome, *m* meters, *PBHS* post-burn hypertrophic scars, *s* seconds, *SAP* sensory action potential, *SCV* sensory conduction velocity, *µV* microVolts

^a^*p* < 0.0001 versus healthy controls

^b^*p* < 0.001 versus healthy controls

^c^*p* < 0.01 versus healthy controls

^d^*p* < 0.05 versus healthy controls

**Table 2:** Summary of motor nerve conduction studies results

	MCV (m/s)		CMAP amplitude (mV)		Latency (ms)	
	Right	Left	Right	Left	Right	Left
Median						
All patients	52.7 ± 4.7^b^	52.9 ± 5.2^b^	9.2 ± 4.1	8.8 ± 4.9	4.2 ± 1.1	4 ± 1^a^
CTS	52.5 ± 3.9^a^	53.1 ± 4.5 ^a^	8.9 ± 3.8	8.6 ± 4.1	4.5 ± 1.1^a^	4.4 ± 1.1^a^
PBHS	53.9 ± 8	54.2 ± 6.5^c^	6.8 ± 3.4	7.9 ± 2.9	3.9 ± 1.9	3.7 ± 0.6^b^
ONP	52.4 ± 1.9^a^	50.9 ± 6.1^a^	10.3 ± 4.4	10.7 ± 7.6^b^	3.3 ± 0.4	3.4 ± 0.3
Healthy controls	57.9 ± 6.3	59.6 ± 5	9.6 ± 4	10.4 ± 5.9	3.2 ± 0.3	3.1 ± 0.4
Ulnar						
All patients	58.6 ± 6.9	59.2 ± 7	8.1 ± 3.1	7 ± 3.4	2.6 ± 0.5	2.7 ± 0.6
CTS	61 ± 5.1	60.2 ± 5.2	10.1 ± 3.9	8.5 ± 2.8	2.4 ± 0.3	2.5 ± 0.5
PBHS	59.1 ± 8	61.1 ± 8	6.4 ± 1.6	7.8 ± 3	2.8 ± 0.5	2.9 ± 0.4
ONP	54.1 ± 6.2	54.1 ± 6.3	6.9 ± 2.3	5.6 ± 4.3	2.6 ± 0.2	2.9 ± 0.8
Healthy controls	59 ± 6.5	58 ± 6.2	9.9 ± 4.1	7.4 ± 3.8	2.7 ± 0.3	3 ± 0.5

*CMAP* compound muscle action potential, *CTS*  Carpal Tunnel Syndrome, *m* = meters, *MCV* motor conduction velocity, *ms* milliseconds, *mV* milliVolts, *ONP* other neuropathic pain, *PBHS* post-burn hypertrophic scars, *s* seconds;

^a^*p* < 0.0001 versus healthy controls

^b^*p* < 0.001 versus healthy controls

^c^*p* < 0.05 versus healthy control

### QST evaluation and sensory phenotype

QST results are summarized in Table 3. The log-transformed CDT values were significantly lower in the left dorsum, left little finger and bilateral index in patients than in controls, and ln-transformed VDT scores were higher in the bilateral index and little finger of patients than healthy controls. No significant differences across sides were noted for log-transformed CDT and HPT and ln-transformed VDT scores in either patients or controls. When comparisons were carried out between hands, painful hands had a lower index and little finger log-transformed CDT than non-painful hands and healthy controls’ hands (and lower dorsum log-transformed CDT values than healthy controls’ hands). The ln-transformed VDT values were higher in painful hands’ little digits than non-painful hands and healthy controls’ hands, and in painful hands’ index fingers than in healthy controls’ hands. HPT was higher in non-painful hands than in painful and healthy controls’ hands. Frequency of different patterns of loss and gain of sensory function at painful sites is different among CTS, PBHS and ONP: L1 is significantly more frequent in PBHS than CTS (8 of 13 patients vs 6 of 34, *p* = 0.01) and ONP (0 of 12, *p* = 0.001) while L3 is more frequent in ONP than PBHS (8 of 12 vs 2 of 13, *p* = 0.01); G2 is significantly more frequent in PBHS than CTS (7 of 13 vs 3 of 34 patients, *p* = 0.002).

**Table 3:** Summary of quantitative sensory testing results and z-scores

Sensory modality	Site	Side	CTS	PBHS	Other neuropathic pain	Healthy controls
CDT (C°)	Dorsum	R	31.1 ± 1.6 [− 3.06 ± 7.57]	28.7 ± 5.9^b^ [− 16.1 ± 35.8]	31.3 8 ± 0.74^c^ [− 1.51 ± 3.14]	31.6 ± 0.2
		L	31.4 ± 0.89 ^a^ [− 2.50 ± 5.59]	27.8 ± 8.4^a^ [− 32.9 ± 80.1]	20.8 ± 14.8 ^b^ [− 275.1 ± 423.8]	31.6 ± 0.2
	II finger	R	29.5 ± 3.8^c^ [− 2.10 ± 4.86]	29.5 ± 3.6^c^ [− 1.92 ± 3.24]	30.6 ± 0.98^c^ [− 0.63 ± 1.1]	31.3 ± 0.6
		L	30.3 ± 2^c^ [− 1.74 ± 3.18]	30.3 ± 1.9 [− 0.45 ± 1.8]	20.7 ± 14.6^b^ [− 88.5 ± 133.9]	31.8 ± 0.9
	V finger	R	30.1 ± 1.84 [− 0.42 ± 1.43]	28.2 ± 5.1 [− 2.01 ± 4.53]	29.9 ± 2.63 [− 0.44 ± 2.01]	30.4 ± 1.3
		L	28.8 ± 3.8^b^ [− 1.91 ± 3.74]	30 ± 1.7 [− 0.61 ± 1.45]	15.7 ± 15.3^b^ [− 63.5 ± 70.7]	30.7 ± 1.7
HPT (C°)	Dorsum	R	43.3 ± 3.9 [− 0.17 ± 1.01]	43.7 ± 4.8 [− 0.03 ± 1.22]	43.6 ± 3.93 [− 0.04 ± 0.97]	43.7 ± 3.9
		L	42.6 ± 3.8 [− 0.40 ± 1.05]	43.6 ± 4.8 [− 0.09 ± 1.35]	44.4 ± 4.61 [0.11 ± 1.25]	43.8 ± 3.5
	II finger	R	46.1 ± 2.7 [0.06 ± 0.75]	45.3 ± 4.3 [− 0.15 ± 1.26]	44.7 ± 3.9 [− 0.34 ± 1.15]	45.9 ± 3.4
		L	44.1 ± 7.6 [− 0.81 ± 4.82]	44.4 ± 5.1 [− 0.29 ± 1.49]	44.8 ± 4.5 [− 0.15 ± 1.27]	45.3 ± 3.5
	V finger	R	45.5 ± 3.3 [− 0.06 ± 0.92]	44.7 ± 3.6 [− 0.32 ± 1.12]	44.7 ± 3.6 [− 0.3 ± 1.12]	45.7 ± 3.5
		L	45.7 ± 3.1 [− 0.06 ± 0.9]	44 ± 4.8 [− 0.58 ± 1.42]	44 ± 3.8 [− 0.27 ± 1.23]	46 ± 3.4
VDT (µm)	II finger	R	1.3 ± 0.92^b^ [0.94 ± 1.14]	0.66 ± 0.6 [0.17 ± 100]	1.41 ± 1.41 ± 0.89 ± 1.27]	0.57 ± 0.46
		L	1.5 ± 1.75^c^ [0.91 ± 1.76]	0.81 ± 0.7 [0.56 ± 1.17]	26.5 ± 50.6^c^ [2.43 ± 3.27]	0.48 ± 0.36
	V finger	R	1.4 ± 1.32^b^ [0.96 ± 1.11]	0.87 ± 1.02^c^ [0.47 ± 0.95]	1.38 ± 2.04 [0.71 ± 1.29]	0.59 ± 0.62
		L	1.3 ± 1.34^b^ [0.94 ± 1.09]	0.79 ± 0.32 [0.74 ± 0.54]	23.2 ± 4.91 [1.58 ± 2.94]	0.51 ± 0.39

*CDT* Cold Pain Threshold, *CTS* carpal tunnel syndrome, *HPT* heat pain threshold, *L* left, PBHS  post-burn hypertrophic scars, R right, *VDT* vibration detection threshold, µm = micrometrs

z-scores are reported in brackets

^a^log transformed or ln transformed value *p* < 0.001 versus healthy controls

^b^log transformed or ln transformed value *p* < 0.01 versus healthy controls

^c^log transformed or ln transformed value *p* < 0.05 versus healthy controls

### Psychological, health quality and social support evaluation

All psychological, health quality and social support evaluations did not differ significantly between CTS, PBHS and ONP. When patients were considered together, they had significantly higher STAI-Y1, BDI-II, TAS-20 overall, TAS-20 F1, TAS-20 F3, and GHQ-12 values than healthy controls (see Table 4).

**Table 4:** Summary of psychological, health quality and social support evaluation

	Combined patients	CTS	PBHS	Other neuropathic pain	Healthy controls
TAS-20 overall	49.4 ± 10^a^	48.8 ± 8.4^c^	51.5 ± 13.9^c^	49.2 ± 10.3	44.4 ± 8.7
TAS-20 F1	17.2 ± 6.2^c^	16.6 ± 5.5	16.6 ± 7.7	18.9 ± 6.4^b^	14.3 ± 5
TAS-20 F2	12.8 ± 4	12.4 ± 3.6	13.7 ± 4.5	13.1 ± 4.4	13.3 ± 3.8
TAS-20 F3	19.2 ± 3.9^a^	19.2 ± 2.8^b^	21.1 ± 5.1^b^	17.4 ± 4.5	16.3 ± 3.8
STAI-Y1	40.2 ± 12.6^c^	37.7 ± 11.5	43.1 ± 18.1	43.6 ± 8.9^a^	34.7 ± 6.8
STAI-Y2	41.3 ± 10.5	39.6 ± 9.4	43.1 ± 15.2	43.9 ± 8^c^	38.7 ± 8.2
BDI-II	11.3 ± 8.8^a^	9.2 ± 7.3^c^	15.2 ± 11.9^c^	12.7 ± 8.5 ^b^	5.6 ± 4.6
GHQ-12	3.2 ± 4.6^c^	1.9 ± 2.3	5.7 ± 7.6	4.1 ± 4.9^c^	1.3 ± 2.0
MSPSS	66.2 ± 18.4	69.2 ± 12	67.6 ± 21.7	57.8 ± 26.1	70.3 ± 11
Family	22.4 ± 5.8	22.9 ± 4.5	22.8 ± 7.1	20.7 ± 7.1	22.6 ± 5.8
Friends	20.1 ± 6.9	20.9 ± 6.1	21 ± 6.1	17.5 ± 9.1	22.8 ± 3.5
Significant other	23.5 ± 5.7	24.7 ± 3.4	23.6 ± 6.4	20.3 ± 8.5	24.1 ± 4.5

*BDI-II* beck depression inventory-II, *CTS* carpal tunnel syndrome, *GHQ-12* 12-item general health questionnairre, *MSPSS* multidimensional scale of perceived social support, *PBHS* post-burn hypertrophic scars, *STAI-Y* form Y of the state-trait anxiety inventory, *TAS-20* toronto alexithymia scale

^a^*p* < 0.001 versus healthy controls

^b^*p* < 0.01 versus healthy controls

^c^*p* < 0.05 versus healthy controls

### Correlation analysis between psychological features

TAS-20 overall correlated with STAI-Y1 (*r* = 0.49, *p* < 0.0001), STAI-Y2 (*r* = 0.53, *p* < 0.0001), BDI-II (*r* = 0.35, *p* = 0.001), TAS-20 F1 (*r* = 0.82, *p* < 0.0001), TAS-20 F2 (*r* = 0.72, *p* < 0.0001) and TAS-20 F3 (*r* = 0.48, *p* < 0.0001). TAS-20 F1 correlated with STAI-Y1 (*r* = 0.55, *p* < 0.0001), STAI-Y2 (*r* = 0.61, *p* < 0.0001), BDI-II (*r* = 0.52, *p* < 0.0001), and GH-12 (*r* = 0.30, *p* = 0.01). The GHQ-12 score correlated with STAI-Y1 (*r* = 0.48, *p* < 0.0001), STAI-Y2 (*r* = 0.54, *p* < 0.0001), BDI-II (*r* = 0.56, *p* < 0.0001). The BDI-II score was significantly correlated with STAI-Y1 (*r* = 0.65, *p* < 0.0001), STAI-Y2 (*r* = 0.75, *p* < 0.0001).

### Correlation analysis between psychological features, QST and neurophysiological findings

In patients, left hand VDT z-scores were significantly correlated with TAS-20 overall (index *r* = 0.38, *p* = 0.004, little finger, *r* = 0.4, *p* = 0.003), TAS-20 F1 (index *r* = 0.42, *p* = 0.001, little finger, *r* = 0.35, *p* = 0.01). Left hand CDT z-scores were correlated with NRS (index *r* = − 0.32, *p* = 0.02; little finger *r* = − 0.38, *p* = 0.01). Left hand HPT z-scores were correlated with STAI-Y1 (dorsum, *r* = 0.34, *p* = 0.01), and STAI-Y2 (dorsum *r* = 0.27, *p* = 0.03). Right hand HPT z-scores were correlated with STAI-Y1 (dorsum, *r* = 0.37, *p* = 0.006). Alexithymic patients had higher VDT z-scores in the left index (mean: 2.98 ± 2.22 vs 1.97 ± 1.56 and 0.31 ± 1.41, *p* = 0.001) and left little digit (mean: 2.85 ± 1.92 vs 0.95 ± 2.08 and 0.70 ± 1.02, *p* = 0.009) than borderline alexithymic and non-alexithymic patients. No significant difference in TAS-20 overall, TAS-20 F1, TAS-20 F2 or TAS-20 F3 was evident between patients with pain only affecting the right hand and those with pain only affecting left hand (respectively, overall 47.8 ± 8.9 vs 52.8 ± 9.1; F1: 17.5 ± 4.8 vs 17.9 ± 5.7; F2: 12.3 ± 4.1 vs 14.1 ± 4.1; F3: 18.1 ± 5.0 vs 20.9 ± 2.5). There was a significant inverse correlation between TAS-20 overall, TAS-20 F1 scores and both the left median (respectively, *r* =  − 0.38, *p* = 0.003; *r* = − 0.28, *p* = 0.03) and left ulnar SAP amplitudes (respectively, *r* = − 0.52, *p* < 0.001; *r* = − 0.38, *p* = 0.03).

When alexithymia was compared according to sensory phenotype, there was an overall difference of TAS-20 F1 among L0, L1, L2 and L3 without any interaction by the diagnostic group (L0 = 13.2 ± 3.5; L1 = 17.7 ± 8; L2 = 18.7 ± 4; L3 = 18.7 ± 6.3, *F* = 1.47, *p* = 0.04). Post hoc analysis showed a significant difference of TAS-20 F1 between L0 and L2/3 (*p* = 0.008).

### General linear model analysis

The GLM analysis revealed a significant relationship among left index VDT z-scores and TAS-20 overall TAS-20 F1 and left median SAP amplitude (respectively, *β* = 33.79, *F* = 10.12, *p* = 0.002; *β* = 28.08, *F* = 11.94, *p* = 0.001; *β* = 23.78, *F* = 10.11, *p* = 0.002). Years of education did not influence the relationship between VDT z-scores and either TAS-20.

## Discussion

Our results demonstrated the association between impairment of vibratory sensation of the left hand, reflecting cutaneous mechanoceptor dysfunction, and alexithymia, particularly the capacity to identify feelings as expressed by the TAS-20 F1 sub-score. This relationship is not dependent on educational level. Importantly, the relationship of alexithymia to sensory impairment is limited only to the left hand. Log-transformed CDT and HPT, and particularly ln-transformed VDT, did not differ significantly between right and left upper limbs, either in healthy controls or in patients. This observation points out that the relationship between alexithymia and sensory impairment of the left hand is not biased by an unequal distribution of sensory abnormalities between the upper limbs in our series. Furthermore, TAS-20 F1 is significantly higher when sensory phenotype is dominated by impairment of vibration or tactile perception then when no sensory impairment is detected.

Therefore, our results may be explained by taking into account the neural correlates of alexithymia and the relationship between perception and emotion. Alexithymia in patients suffering from major depression is associated with a decreased anatomical and functional connectivity between left and right sensorimotor cortices through the corpus callosum, while alexithymia without concurrent major depression is associated with altered diffusivity of the subcomponent of the right superior longitudinal fascicle that anatomically connects the prefrontal cortex and superior occipital lobe (Ho et al. 2016). This points to a prominent association of alexithymia with dysfunction in the right hemisphere, which is important for emotional processing, as well as an impairment in the transfer of information between the emotional-dominant and language-dominant hemispheres (Ho et al. 2016; Donges and Suslow 2017). The relationship between difficulty in identifying feelings and VDT is in keeping with the involvement of emotions in touch perception (Gazzola et al. 2012; Ravaja et al. 2017). Primary somatosensory cortex activation during interpersonal touch is modulated by the facial expression and gender of the touching person, as shown by fMRI (Gazzola et al. 2012) and somatosensory-evoked potential results data (Ravaja et al. 2017). Recently, TAS-20 overall, TAS-20 F1 and TAS-20 F2 scores have been related to warm perception thresholds in the left upper limb of healthy subjects (Borhani et al. 2017). These authors did not log-transform the QST results or evaluate z-scores, in contrast to the suggestions of the German Neuropathic Pain Research Network (DFNS) (Geber et al. 2011; Rolke et al. 2006). Furthermore, warmth detection thresholds were evaluated using a reaction-time-dependent method of limits. In our study, we did not evaluate warmth detection thresholds, and VDT was evaluated using a reaction-time-independent method of levels. Despite these discrepancies, both our study and that of Borhani et al (2017) suggest that alexithymia may be associated with processing of sensory inputs from the left of upper limbs, even though differently in patients and controls.

Besides alexithymia, in our study, different sensory modalities evaluated by QST were also correlated with pain intensity ratings (CDT), as well as both state and trait anxiety (HPT), further supporting the association between emotional processing and perception.

Taken together, these observations suggest that psychological features, including emotional processing, could be interpreted also taking into account QST results and NCS. QST has been evaluated as a potential biomarker for neuropathic and non-neuropathic pain, and may help to identify a possible pathophysiological mechanism underlying pain in a patient, thus guiding treatment (Smith et al. 2017; Treede 2019). Both a subject’s sensory phenotype (Treede 2019) and alexithymia (Valdespino et al. 2017) are considered trans-diagnostic features (i.e. occurring in patients affected by different diseases, but probably stemming from a common pathophysiology). In our study, alexithymia did not differ among patients with CTS, ONP and PBHS, but did correlate with a subject’s vibration threshold as a measure of loss of mechanoceptor function.

In our study, we did not find any relationship between alexithymia and pain intensity: NRS was correlated with CDT, in keeping with previous reports suggesting the critical role of small fibers in the pathophysiology of neuropathic pain (Truini et al. 2009). The association of dorsum of hands HPT with state anxiety may be related to the higher thermo-sensitivity of hairy proximal skin in the hands (Filingeri et al., 2018) and the known role of hairy skin thermal receptors in body thermo-regulation. Anxiety and thermoregulation are linked (Adriaan Bouwknecht et al., 2007). Heat stimuli are conveyed to the lateral parabrachial nucleus which in turn influence the preoptic hypothalamus for thermoregulation (Tansey and Johnson 2015) and the central nucleus of the amygdale (Cai et al. 2018). Optogenic activation of the latter projection induces acute anxiety-like behavior in mice (Cai et al. 2018).

In contrast, alexithymia was correlated with VDT and ulnar and median SAP amplitude, thus supporting a distinct mechanism. The association between the difficulty identifying feelings and measure of psychological distress, which is not the case for pain intensity, is interesting and may warrants further investigation.

Taken together, our results suggest a relationship between sensory impairment expressed by QST and NCS results and alexithymia and perceived social support, and that alexithymia is related to health quality in patients with neuropathic pain, independently of the intensity of pain itself.

### Limitations

This study has some limitations that should be considered. First, we used self-reported instruments for the assessment of psychological, health quality, and social support variables. With regard to alexithymia, this may have led to an underestimation of the presence of participants with alexithymia, in particular of individuals who scored close to the cut-off scores. Structured interviews, less dependent on the individuals’ awareness levels, should be used, in addition to the traditional TAS-20 questionnaire. Second, we adopted a cross-sectional design, which does not allow us to draw firm conclusions with regard to the causality of the emergent relationships. Third, even though we enrolled an adequate number of participants, our study is limited by a relatively small number of participants, and future studies should recruit a larger number of participants. Fourth, we found an association between alexithymia and both VDT and sensory phenotype with prominent loss of mechanoceptor function, but further studies are needed to evaluate a possible causal relationship.

## Conclusion

Despite the limitations described, the current study sheds new light on the correlation between sensory phenotype and alexithymia in patients with neuropathic pain in the upper limbs. Based on these results, we suggest the importance of delivering to these patients personalized care that takes into account, from a biopsychosocial perspective, not only the neurophysiological aspects but also aspects of their mental functioning, especially with regard to their capacity to identify and describe feelings.

## References

[CR1] Adriaan Bouwknecht J, Olivier B, Paylor RE (2007). The stress-induced hyperthermia paradigm as physiological animal model for anxiety: a review of pharmacological anxiety model in the mouse. Neurosci Biobehav Rev.

[CR2] American Academy of Neurology (1993). Practice parameters for carpal tunnel syndrome. Neurology.

[CR3] Backonja MM, Attal N, Baron R, Bouhassira D, Drangholt M, Dyck PJ, Edwards RR, Freeman R, Gracely R, Haanpaa MH, Hansson P, Hatem SM, Krumova EK, Jensen TS, Maier C, Mick G, Rice AS, Rolke R, Treede RD, Serra J, Toelle T, Tugnoli V, Walk D, Walalce MS, Ware M, Yarnitsky D, Ziegler D (2013). Value of quantitative sensory testing in neurological and pain disorders: NeuPSIG consensus. Pain.

[CR4] Bagby RM, Parker JDA, Taylor GJ (1994). The 20-item Toronto Alexithymia Scale, I: Item selection and cross-validation of the factor structure. J Psychosom Res.

[CR5] Bagby RM, Parker JDA, Taylor GJ (1994). The 20-item Toronto Alexithymia Scale, II: Convergent, discriminant, and concurrent validity. J Psychosom Res.

[CR6] Baliki MN, Apkarian AV (2015). Nociception, pain, negative moods, and behavior selection. Neuron.

[CR7] Baron R, Maier C, Attal N, Binder A, Bouhassira D, Cruccu G, Finnerup NB, Haanpää M, Hansson P, Hüllemann P, Jensen TS, Freynhagen R, Kennedy JD, Magerl W, Mainka T, Reimer M, Rice AS, Segerdahl M, Serra J, Sindrup S, Sommer C, Tölle T, Vollert J, Treede RD (2017). Peripheral neuropathic pain: a mechanism-related organizing principle based on sensory profiles. Pain.

[CR8] Beck AT, Steer RA, Brown GK (1996). Beck depression inventory.

[CR9] Borhani K, Làdavas E, Fotopoulou A, Haggard P (2017). “Lacking warmth”: Alexithymia trait is related to warm-specific thermal somatosensory processing. Biol Psychol.

[CR10] Bouhassira D, Attal N, Alchaar H, Boureau F, Brochet B, Bruxelle J, Cunin G, Fermanian J, Ginies P, Grun-Overdyking A, Jafari-Schluep H, Lantéri-Minet M, Laurent B, Mick G, Serrie A, Valade D, Vicaut E (2005). Comparison of pain syndromes associated with nervous or somatic lesions and development of a new neuropathic pain diagnostic questionnaire (DN4). Pain.

[CR11] Cai YQ, Wang W, Paulucci-Holthauzen A, Pan ZZ (2018). Brain circuits mediating opposing effects on emotion and pain. J Neurosci.

[CR12] Di Tella M, Adenzato M, Catmur C, Miti F, Castelli L, Ardito RB (2020). The role of alexithymia in social cognition: Evidence from a non-clinical population. J Affect Disord.

[CR13] Di Tella M, Castelli L (2016). Alexithymia in chronic pain disorders. Curr Rheumatol Rep.

[CR14] Di Tella M, Enrici I, Castelli L, Colonna F, Fusaro E, Ghiggia A, Romeo A, Tesio V, Adenzato M (2018). Alexithymia, not fibromyalgia, predicts the attribution of pain to anger-related facial expressions. J Affect Disord.

[CR15] Donges US, Suslow T (2017). Alexithymia and automatic processing of emotional stimuli: a systematic review. Rev Neurosci.

[CR16] Ferrante MA (2004). Brachial plexopathies: classification, causes and consequences. Muscle Nerve.

[CR17] Filingeri D, Zhang H, Arens EA (2018). Thermosensory micromapping or warm and cold sensitivity across glabrous and hairy skin of male and female hands and feet. J Appl Physiol.

[CR18] Finnerup NB, Haroutounian S, Kamerman P, Baron R, Bennett DL, Bouhassira D, Cruccu G, Freeman R, Hansson P, Nurmikko T, Raja SN, Rice AS, Serra J, Smith BH, Treede RD, Jensen TS (2016). Neuropathic pain: an updated grading system for research and clinical practice. Pain.

[CR19] Gazzola V, Spezio ML, Etzel JA, Castelli F, Adolphs R, Keysers C (2012). Primary somatosensory cortex discriminates affective significance in social touch. Proc Natl Acad Sci USA.

[CR20] Geber C, Klein T, Azad S, Birklein F, Gierthmühlen J, Huge V, Lauchart M, Nitzsche D, Stengel M, Valet M, Baron R, Maier C, Tölle T, Treede RD (2011). Test-retest and interobserver reliability of quantitative sensory testing according to the protocol of the German Research Network on Neuropathic Pain (DFNS): a multi-centre study. Pain.

[CR21] Goldberg D, Williams P (1988). A user’s guide to the General Health Questionnaire.

[CR22] Goslin KL, Krivikas LS, Logigian EL (1999). Proximal neuropathies of the upper extremity. Neurologic clinics. Entrapment and other focal neuropathies.

[CR23] Ho NS, Wong MM, Lee TM (2016). Neural connectivity of alexithymia: Specific association with major depressive disorder. J Affect Disord.

[CR24] Hosoi M, Molton IR, Jensen MP, Ehde DM, Amtmann S, O’Brien S, Arimura T, Kubo C (2010). Relationships among alexithymia and pain intensity, pain interference, and vitality in persons with neuromuscular disease: Considering the effect of negative affectivity. Pain.

[CR25] International Association for the study of Pain, terminology [electronic source] www.iasp-pain.org. Accessed 9 Mar 2020.

[CR26] Isoardo G, Stella M, Cocito D, Risso D, Migliaretti G, Cauda F, Palmitessa A, Faccani G, Ciaramitaro P (2012). Neuropathic pain in post-burn hypertrophic scars: a psychophysical and neurophysiological study. Muscle Nerve.

[CR27] Jablecki CK, Andary MT, Floeter MK, Miller RG, Quartly CA, Vennix MJ, Wilson JR (2002). Practice parameter: electrodiagnostic studies in carpal tunnel syndrome: report of the American Academy of Electrodiagnostic Medicine, American Academy of Neurology and the American Academy of Physical Medicine and Rehabilitation. Neurology.

[CR28] Jensen MP, McFarland CA (1993). Increasing the Reliability and validity of pain intensity measurement in chronic pain patients. Pain.

[CR29] Keefer KV, Taylor GJ, Parker JD, Bagby RM (2019). Taxometric analysis of the Toronto Structured Interview for Alexithymia. Assessment.

[CR30] Kendall FP, Kendall McCreary E, Geise Provance P, Rodgers MM, Romani WA (2005). Muscles, testing and function, with posture and pain.

[CR31] Lane RD, Weihs KL, Herring A, Hishaw A, Smith R (2015). Affective agnosia: expansion of the alexithymia construct and a new opportunity to integrate and extend Freud’s legacy. Neurosci Biobehav Rev.

[CR32] Lumley MA, Neely LC, Burger AJ (2007). The assessment of alexithymia in medical settings: implications for understanding and treating health problems. J Pers Assess.

[CR33] Maier C, Baron R, Tölle TR, Binder A, Birbaumer N, Birklein F, Gierthmühlen J, Flor H, Geber C, Huge V, Krumova EK, Landwehrmeyer GB, Magerl W, Maihöfner C, Richter H, Rolke R, Scherens A, Schwarz A, Sommer C, Tronnier V, Uçeyler N, Valet M, Wasner G, Treede RD (2010). Quantitative sensory testing in the German Research Network on Neuropathic Pain (DFNS): somatosensory abnormalities in 1236 patients with different neuropathic pain syndromes. Pain.

[CR34] Margalit D, Ben Har L, Brill S, Vatine JJ (2014). Complex regional pain syndrome, alexithymia, and psychological distress. J Psychosom Res.

[CR35] Makino S, Jensen MP, Arimura T, Obata T, Anno K, Iwaki R, Kubo C, Sudo N, Hosoi M (2013). Alexithymia and chronic pain: the role of negative affectivity. Clin J Pain.

[CR36] Meints SM, Edwards RR (2018). Evaluating psychosocial contributions to chronic pain outcomes. Prog Neuropsychopharmacol Biol Psychiatry.

[CR37] Mondelli M, Giannini F, Ballerini M, Ginanneschi F, Martorelli E (2005). Incidence of ulnar neuropathy at the elbow in the province of Siena (Italy). J Neurol Sci.

[CR38] Nicholas M, Vlaeyen JWS, Rief W, Barke A, Aziz Q, Benoliel R, Cohen M, Evers S, Giamberardino MA, Goebel A, Korwisi B, Perrot S, Svensson P, Wang SJ, Treede RD (2019). The IASP classification of chronic pain for ICD-11: chronic primary pain. Pain.

[CR39] Padua L, Pasqualetti P, Rosenbaum R (2005). One patient, two carpal tunnels: statistical and clinical analysis- by hand or by patient?. Clin Neurophysiol.

[CR40] Perrot S, Cohen M, Barke A, Korwisi B, Rief W, Treede RD (2019). The IASP classification of chronic pain for ICD-11: Chronic secondary musculoskeletal pain. Pain.

[CR41] Preston DC, Logigian EL (1999). Distal median neuropathies. Neurologic clinics. Entrapment and other focal neuropathies.

[CR42] Ravaja N, Harjunen V, Ahmed I, Jacucci G, Spapé MM (2017). Feeling touched: emotional modulation of somatosensory potentials to interpersonal touch. Sci Rep.

[CR43] Rolke R, Baron R, Maier C, Tölle TR, Treede RD, Beyer A, Binder A, Birbaumer N, Birklein F, Bötefür IC, Braune S, Flor H, Huge V, Klug R, Landwehrmeyer GB, Magerl W, Maihöfner C, Rolko C, Schaub C, Scherens A, Sprenger T, Valet M, Wasserka B (2006). Quantitative sensory testing in the German Research Network on Neuropathic Pain (DFNS): Standardised protocol and reference values. Pain.

[CR44] Rosenberger DC, Blechschmidt V, Timmerman H, Wolff A, Treede RD (2020). Challenges of neuropathic pain: focus on diabetic neuropathy. J Neural Transm.

[CR45] Saariaho AS, Saariaho TH, Mattila AK, Karukivi M, Joukamaa MI (2015). Alexithymia and early maladaptive schemas in chronic pain patients. Scand J Psychol.

[CR46] Scholz J, Finnerup NB, Attal N, Aziz Q, Baron R, Bennett MI, Benoliel R, Cohen M, Cruccu G, Davis KD, Evers S, First M, Giamberardino MA, Hansson P, Kaasa S, Korwisi B, Kosek E, Lavandʼhomme P, Nicholas M, Nurmikko T, Perrot S, Raja SN, Rice ASC, Rowbotham MC, Schug S, Simpson DM, Smith BH, Svensson P, Vlaeyen JWS, Wang SJ, Barke A, Rief W, Treede RD (2019). The IASP classification of chronic pain for ICD-11: chronic neuropathic pain. Pain.

[CR47] Schug SA, Lavandʼhomme P, Barke A, Korwisi B, Rief W, Treede RD, IASP Taskforce for the Classification of Chronic Pain (2019). The IASP classification of chronic pain for ICD-11: chronic postsurgical or posttraumatic pain. Pain.

[CR48] Seror P (2002). The medial antebrachial cutaneous nerve: antidromic and orthodromic conduction studies. Muscle Nerve.

[CR49] Sifneos PE (1973). The prevalence of 'alexithymic' characteristics in psychosomatic patients. Psychother Psychosom.

[CR50] Smith SM, Dworkin RH, Turk DC, Baron R, Polydefkis M, Tracey I, Borsook D, Edwards RR, Harris RE, Wager TD, Arendt-Nielsen L, Burke LB, Carr DB, Chappell A, Farrar JT, Freeman R, Gilron I, Goli V, Haeussler J, Jensen T, Katz NP, Kent J, Kopecky EA, Lee DA, Maixner W, Markman JD, McArthur JC, McDermott MP, Parvathenani L, Raja SN, Rappaport BA, Rice ASC, Rowbotham MC, Tobias JK, Wasan AD, Witter J (2017). The potential role of sensory testing, skin biopsy, and functional brain imaging as biomarkers in chronic pain clinical trials: IMMPACT Considerations. J Pain.

[CR51] Spielberger CD, Gorsuch RL, Lushene R, Vagg PR, Jacobs GA (1983). State-trait anxiety inventory (form Y).

[CR52] Tansey EA, Johnson CD (2015). Recent advances in thermoregulation. Adv Physiol Educ.

[CR53] Treede RD (2019). The role of quantitative sensory testing in the prediction of chronic pain. Pain.

[CR54] Truini A, Padua L, Biasiotta A, Caliandro P, Pazzaglia C, Galeotti F, Inghilleri M, Cruccu G (2009). Differential involvement of A-delta and A-beta fibres in neuropathic pain related to carpal tunnel syndrome. Pain.

[CR55] Turk DC, Audette J, Levy RM, Mackey SC, Stanos S (2010). Assessment and treatment of psychosocial comorbidities in patients with neuropathic pain. Mayo Clin Proc.

[CR56] Vachon-Presseau E, Centeno MV, Ren W, Berger SE, Tétreault P, Ghantous M, Baria A, Farmer M, Baliki MN, Schnitzer TJ, Apkarian AV (2016). The emotional brain as a predictor and amplifier of chronic pain. J Dent Res.

[CR57] Valdespino A, Antezana L, Ghane M, Richey JA (2017). Alexithymia as a transdiagnostic precursor to empathy abnormalities: the functional role of the insula. Front Psychol.

[CR58] Wade RG, Takwoingi Y, Wormald JCR, Ridgway JP, Tanner S, Rankine JJ, Bourke G (2018). Magnetic resonance imaging for detecting root avulsions in traumatic adult brachial plexus injuries: protocol for a systematic review of diagnostic accuracy. Syst Rev.

[CR59] Zimet GD, Dahlem N, Zimet SG, Farley GK (1988). The multidimensional scale of perceived social support. J Pers Assess.

